# Role of Tetraspanins in Hepatocellular Carcinoma

**DOI:** 10.3389/fonc.2021.723341

**Published:** 2021-09-03

**Authors:** Sicheng Cai, Yue Deng, Huiming Peng, Jian Shen

**Affiliations:** ^1^Department of Gastroenterology, Union Hospital, Tongji Medical College, Huazhong University of Science and Technology, Wuhan, China; ^2^Department of Human Anatomy, School of Basic Medicine, Tongji Medical College, Huazhong University of Science and Technology, Wuhan, China; ^3^Cancer Center, Union Hospital, Tongji Medical College, Huazhong University of Science and Technology, Wuhan, China; ^4^Department of Pancreatic Surgery, Union Hospital, Tongji Medical College, Huazhong University of Science and Technology, Wuhan, China

**Keywords:** hepatocellular carcinoma, tetraspanin, tetraspanin family, tumor proliferation, tumor metastasis, targeted therapy, drug resistance

## Abstract

Hepatocellular carcinoma (HCC) is characterized by high prevalence, morbidity, and mortality. Liver cancer is the sixth most common cancer worldwide; and its subtype, HCC, accounts for nearly 80% of cases. HCC progresses rapidly, and to date, there is no efficacious treatment for advanced HCC. Tetraspanins belong to a protein family characterized by four transmembrane domains. Thirty-three known tetraspanins are widely expressed on the surface of most nucleated cells and play important roles in different biological processes. In our review, we summarize the functions of tetraspanins and their underlying mechanism in the life cycle of HCC, from its initiation, progression, and finally to treatment. CD9, TSPAN15, and TSPAN31 can promote HCC cell proliferation or suppress apoptosis. CD63, CD151, and TSPAN8 can also facilitate HCC metastasis, while CD82 serves as a suppressor of metastasis. TSPAN1, TSPAN8, and CD151 act as prognosis indicators and are inversely correlated to the overall survival rate of HCC patients. In addition, we discuss the potential of role of the tetraspanin family proteins as novel therapeutic targets and as an approach to overcome drug resistance, and also provide suggestions for further research.

## 1 Introduction

Liver cancer was the sixth most common cancer worldwide in 2018, with an annual death toll of over 782,000. Hepatocellular carcinoma (HCC), also known as hepatoma, constitutes nearly 80% of liver cancer cases and poses a significant burden on global healthcare systems due to its prevalence and high death rate (1). A survey in 2012 revealed that HCC in developing countries accounted for 8.1% of all new cancer cases and 83% of all HCC cases worldwide (2). HCC is also the second most common cause of cancer-related death (2). Chronic liver diseases such as chronic hepatitis B and C, among others, are significant risk factors for HCC (3, 4).

Tetraspanins are a family of transmembrane proteins with four transmembrane domains (TM1, TM2, TM3, and TM4), two extracellular loops (ECL1 and ECL2), and one intracellular loop. ECL2 is essential to the functions of tetraspanins since most of protein–protein interaction sites have been mapped to ECL2. ECL2 consists of a constant domain and a variable domain. While the constant domain facilitates interactions between different tetraspanin molecules, the variable domain accounts for interactions with other non-tetraspanin proteins. The conserved cysteine residues in ECL2 can form a disulfide bond within the same ECL2. TM domains contain many polar residues that can stabilize tetraspanin structure with the help of ECL2 disulfide crosslinks. The structure and function of ECL1 have so far remained unclear (5, 6).

Tetraspanins are widely expressed in metazoans. In *Homo sapiens*, the tetraspanin family has 33 family members (7). Some tetraspanin-like proteins have also been identified in plants (8). Since 65% to 95% of amino acids are highly conserved among the tetraspanin family, it is likely that they share similar functions across different species (9).

Tetraspanins are expressed on the surface of most nucleated cells (10) and play important roles in cell proliferation, differentiation, adhesion, migration, and cell–cell crosstalk (7–11). In terms of tumor biology, tetraspanins are indispensable at all the stages of tumor initiation and progression, showing both tumor-promoting and tumor-inhibiting functions (5, 10, 12–14). Specifically, tetraspanins are involved in viral infection, such as hepatitis virus C (HCV), which is a high-risk carcinogenesis factor for HCC (15). Indeed, recent studies on HCC pathogenesis have accrued evidences to support the vital roles of tetraspanins in HCC development (5, 13).

Tetraspanins take part in different biological processes mainly *via* interacting with different partner molecules. Integrins are the most prominent partner of tetraspanins (5, 13). Through binding to integrins, tetraspanins can influence the distribution and function of integrins. Tetraspanins mainly act as a modulator by controlling the trafficking of their partner (8). Tetraspanins can also make an impact on their partners directly by influencing the function of their partner, without influencing the trafficking of their partner. For instance, CD151 can enhance integrin-mediated adhesion to laminin, resulting in integrin signaling activation (16). CD151 can form a complex with integrin α3β1 and regulate PI3K or PI4K signaling pathway in different cancer cells, which finally impacts cancer cell migration *via* rearrangement of actin cytoskeleton or metalloproteinase secretion. In melanoma cell line, CD151–α3β1/α6β4 integrin complexes recruit small G proteins (Ras, Rac1, and Cdc42) to form integrin–CD151–GTPase complexes, finally leading to GTPase activation (17). Besides integrins, tetraspanins can also form a complex with various other proteins such as G protein-coupled receptors (GPCRs), G proteins, growth factor receptors, or some non-proteins such as cholesterol and gangliosides to form tetraspanin-enriched membrane microdomains (TEMs). They are involved in the crosstalk between growth factor receptors and integrins, mediating growth factor pathway such as hepatocyte growth factor receptor (HGFR) signaling pathway, epidermal growth factor receptor (EGFR) signaling pathway, and transforming growth factor-β (TGF-β) signaling pathway (18).

We herein present the interaction between tetraspanins and HCC and their potential as targets for HCC treatment.

## 2 Tetraspanins as HCC Risk Factors

Hepatitis B virus (HBV), HCV, cirrhosis, hereditary hemochromatosis, and non-alcoholic fatty liver disease are all proven risk factors of HCC (3). Tetraspanins are associated with HBV, HCV infection, and cirrhosis and therefore may play a vital role in precancerous disease as well as HCC initiation.

### 2.1 Role of Tetraspanins in Hepatitis B-Associated HCC

The incidence of HCC positively correlates with serum levels of HBV DNA, which is a marker of HBV infection and viral proliferation. For individuals with a serum level of HBV DNA above 1 million copies per milliliter, the cumulative incidence of HCC could reach as high as 14.9% (19). CD82 is a member of the tetraspanin family and performs a variety of functions in HCC progression and metastasis. CD82 is the only tetraspanin that is proven to be a suppressor of HCC progression. In HCC cell lines, Yu et al. found that HBx, a component of HBV, could induce CD82 promoter methylation and impair CD82 expression at the transcriptional level (20). However, an analysis of clinical samples failed to reveal any statistically significant differences in CD82 expression between HBsAg-positive and HBsAg-negative samples. Nevertheless, the HBV-induced downregulation of CD82 may accelerate HCC progression.

### 2.2 Role of Tetraspanins in Hepatitis C-Correlated HCC

HCV infection is a critical risk factor for HCC. A prospective cohort study in China reported that 23.73% of HCV-positive men and 16.71% of HCV-positive women may develop HCC during their lifetime (21). CD81, a tetraspanin, binds to E2, the HCV envelop protein, *via* its ECL2 domain and facilitates the entry of HCV into hepatocytes. CD81 also mediates immune reactions against viral infections by inducing the secretion of IFNα in the infected cells. CD81 and CD9 are involved in the recognition of HCV-infected cells by plasmacytoid dendritic cells (pDCs), while direct cell–cell contact is a vital step of IFN induction (22).

### 2.3 Role of Tetraspanins in Cirrhosis-Associated HCC

Almost all chronic liver diseases cause liver fibrosis. Although reversible at its initial stage, liver fibrosis can eventually progress into cirrhosis, which is a known risk factor of HCC (23, 24).

#### 2.3.1 TSPAN5

TSPAN5 is a tetraspanin that regulates ADAM10, a metalloprotease, and thereby activates Notch signaling (25). The prognosis of patients with cirrhosis correlates with the epigenetic modification of TSPAN5. Lubecka et al. reported that among HBV-negative cirrhosis patients, hypomethylation of TSPAN5 gene is more frequently found in the patients who eventually develop HCC (26). Therefore, the locus-specific DNA methylation may be a useful biomarker for screening at-risk populations, and the expression TSPAN5 may be an indicator for carcinogenesis.

#### 2.3.2 CD151

CD151, another tetraspanin, mediates lymphocyte recruitment during the initiation and progression of chronic inflammation. In patients with chronic liver diseases, the upregulation of CD151 is predominantly on hepatic sinusoidal endothelial cells (HSECs) and neovessels, which in turn upregulates the expression of endothelial adhesion molecule/immunoglobulin superfamily member VCAM-1 and subsequently promote lymphocyte adhesion (27).

## 3 Role of Tetraspanins in HCC Cell Growth and Survival

**Figure 1:**
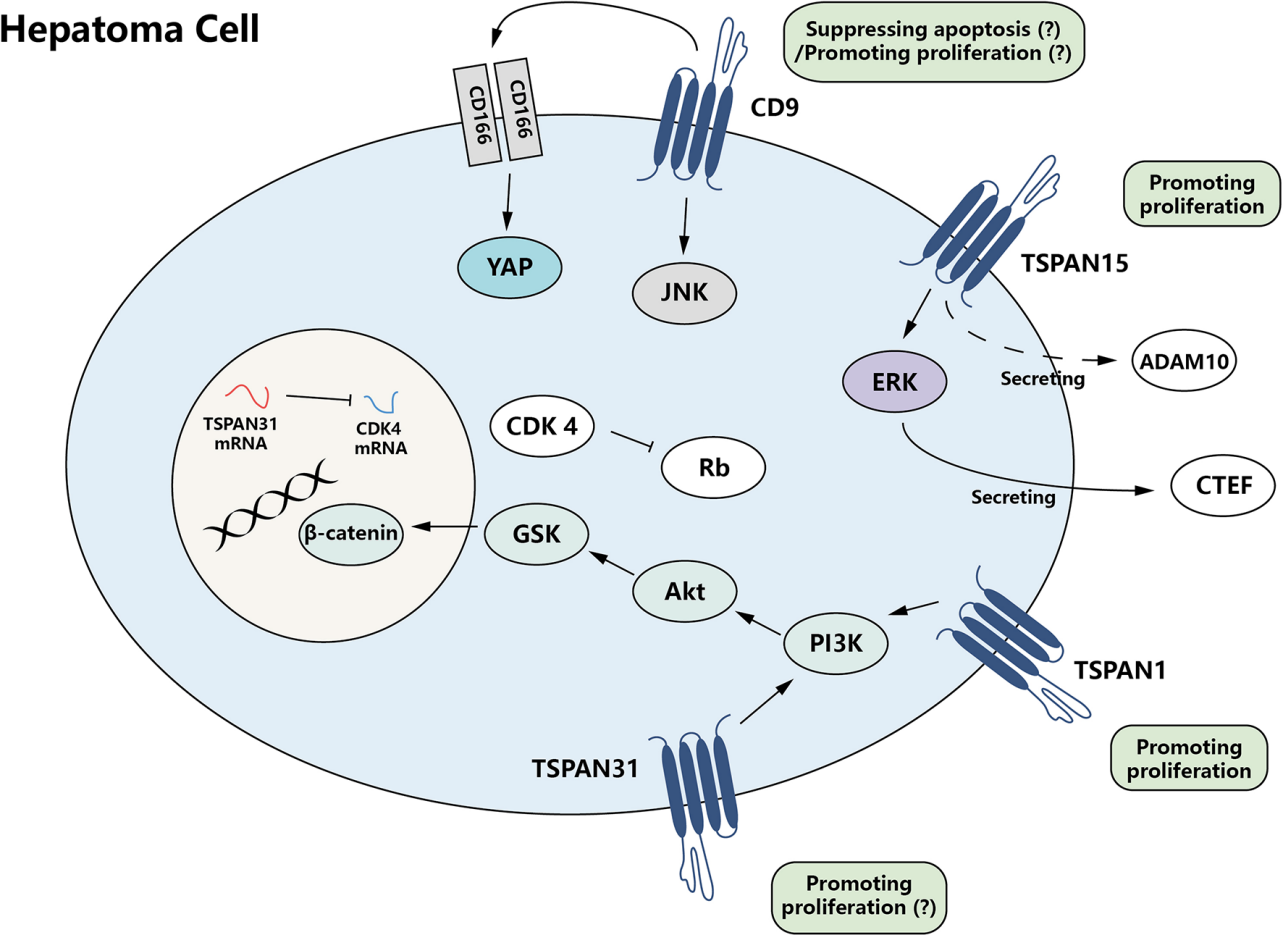
The growth promotion effect of tetraspanin protein family for hepatoma cells. CD9, TSPAN15, and TSPAN31, members of the tetraspanin family, are able to mediate proliferation-promoting and apoptosis-suppressing signals directly or indirectly, which results in modulation of tumor cell growth.

The tetraspanin family facilitates hepatoma promotion by activating proliferation and anti-apoptotic properties of HCC cells. Some tetraspanins, such as TSPAN15, can enhance tumor cell proliferation ability (28), while other family members, such as CD9, CD63, TSPAN1, TSPAN7, and TSPAN31, can antagonize apoptosis and facilitate tumor cell survival (29–31). These two effects act together in promoting the development of tumors (**Figure 1**).

### 3.1 TSPAN15

TSPAN15 exhibits a heterogeneous expression pattern in different HCC patients. In some specific types of HCC, the level of TSPAN15 is upregulated, which positively associates with the stemness of cancer cells and recurrence of cancer significantly (28). Stemness is the core property of stem cells, including self-renewal and the ability to generate differentiated progeny (32). Sidahmed-Adrar et al. found that TSPAN15 could enhance the phosphorylation of ERK, which controls the expression and secretion of connective tissue growth factor (CTGF) and thereby promotes HCC cell proliferation. The pro-proliferation effect may also relate to that TSPAN15 can enhance the metalloprotease ADAM10 secretion to some extent, but with unclear mechanism. TSPAN15 expression is also associated with several stemness markers such as epithelial cell adhesion molecule (EpCAM) and cytokeratin-19 (CK19) (28).

### 3.2 CD9

CD9 activity alone does not suppress hepatoma cell apoptosis, but it is believed that it can enhance the function of CD166 (33), thus contributing to the survival of HCC cells indirectly. CD166 can promote the transcription of Yes-associated Protein (YAP), a critical anti-apoptosis effector in HCC (34), *via* cAMP-response element-binding protein (CREB) (29). CD166 mainly functions as a CD166–CD166 or CD166–CD6 dimer (33), and CD9 can facilitate CD166–CD166 homophilic interaction, which accentuates CD166 anti-apoptosis effect at the protein level (29).

Paradoxically, CD9 itself displays an opposing function in a recent study. It is shown that CD9 can inhibit HCC cell proliferation *in vitro* and that knockdown of CD9 enhances HCC tumorigenicity *in vivo* (35). CD9 can inhibit the phosphorylation and promoter activity of JNK and c-JUN. Therefore, downregulation of CD9 in HCC can finally lead to JNK signaling pathway activation, which promotes HCC cell proliferation *via* downstream factors cyclin D1 and Bcl-2. It is hard to explain how CD9 can perform contradictory functions. Whether CD9 acts as a suppressor of HCC cell apoptosis or proliferation under different conditions still needs further investigation. What is currently known is that Krüppel-like factor 4 (KLF4), an important inhibitor of tumor growth in various kinds of cancers, is a transcriptional factor of CD9 (35).

### 3.3 TSPAN1

TSPAN1, commonly known as neuroepithelial transforming gene 1 (NET-1), is a guanine nucleotide exchange factor (GEF) (36). It is upregulated in HCC tissue (37, 38) and identified as a proliferation promoter (39–42). Sun et al. found that after TSPAN1 knockdown, the expression level of some important factors in PI3K/Akt signaling pathway, such as pPI3K, was also decreased, suggesting that TSPAN1 may regulate the proliferation of HCC cells by targeting PI3K/Akt signaling pathway (41).

Based on the fact that TSPAN1 is highly expressed in HCC cells and positively regulating HCC cell proliferation, Wu et al. developed a gene therapy method to specifically silence TSPAN1 gene expression in HCC cells (39, 40, 42, 43). They used targeted nanobubbles to deliver TSPAN1 siRNA to HCC cells, with the aid of ultrasound exposure to increase transfection efficiency. This method could significantly reduce the expression level of TSPAN1 in HCC tissue and prolong the survival interval in mouse model. Considering the role of TSPAN1 in HCC cell proliferation, this gene therapy method can efficiently deliver the genes to cancer cells, which may be one kind of important precision treatment for HCC in the future.

### 3.4 TSPAN7

TSPAN7 transcription and expression level are proven to be decreased in different HCC cell lines, and its overexpression can inhibit cell proliferation *in vivo* (44). However, the mechanism involved in the role of TSPAN7 is not investigated. Despite this phenomenon, either its upstream or downstream crosstalk is still unclear.

### 3.5 TSPAN31

Some evidence suggests that TSPAN31 can also regulate survival and apoptotic signals in HCC cells. TSPAN31 can activate the Akt/GSK-3β/β-catenin signaling pathway, an important pathway for cell survival. However, TSPAN31 mRNA serves as a natural anti-sense transcript of cyclin-dependent kinase 4 (CDK4), a kinase responsible for the phosphorylation of retinoblastoma (Rb) protein, critical in cell cycle regulation. TSPAN31 itself is negatively regulated by tumor suppressor protein p53 (30). Overall, the effects of TSPAN31 on HCC cell proliferation are not significant since TSPAN31 knockdown shows no influence on HCC cell proliferation (30). This may be due to the fact that TSPAN31 exerts bidirectional activity on different pro-proliferation factors simultaneously. Meanwhile, CD63, besides its role in the metastasis of hepatoma (described below), can also favor the survival of hepatoma cells (31).

## 4 Role of Tetraspanins HCC Neoangiogenesis

In order to fulfill the high nutrition requirements of tumor cells, tumors often secrete kinds of pro-angiogenic factors or express some membrane-bound proteins that can contribute to neoangiogenesis. CD151 and TSPAN8 are proteins that participate in vascular formation (45, 46). Anti-angiogenesis has already been a key target of cancer therapy, and several drugs have been proven to be effective and adopted in clinical applications (47). Thus, the close relationship between the tetraspanin family and tumor neoangiogenesis, CD151, and TSPAN8 may represent potential therapeutic targets of HCC in the future.

### 4.1 CD151

CD151 is unique membrane-bound pro-angiogenic factor highly expressed by HCC cells. Its pro-angiogenic effects have been proven by different laboratories both *in vitro* and *in vivo* (45, 48). The pro-angiogenic effects of CD151 are a result of metalloproteinase 9 (MMP9) secreted by high-CD151 expression HCC cells. As mentioned above, possibly by forming heterodimer receptors with integrin and binding laminin 5 (49), CD151 can simulate downstream PI3K/Akt/GSK-3b/Snail signaling pathway to control MMP9 expression (48). Besides, MMP9 also plays a role in HCC metastasis (as follows).

### 4.2 TSPAN8

Similar to CD151, human umbilical vein endothelial cells (HUVECs) were co-cultured with TSPAN8 knockdown HCC cells to reduce HUVEC tube formation, which is an indicator of low angiogenic activity, when compared with HUVEC cells co-cultured with control HCC cells (46). This reveals that TSPAN8 may also promote neoangiogenesis of unclear mechanism.

## 5 Role of Tetraspanin in HCC Metastasis

**Figure 2:**
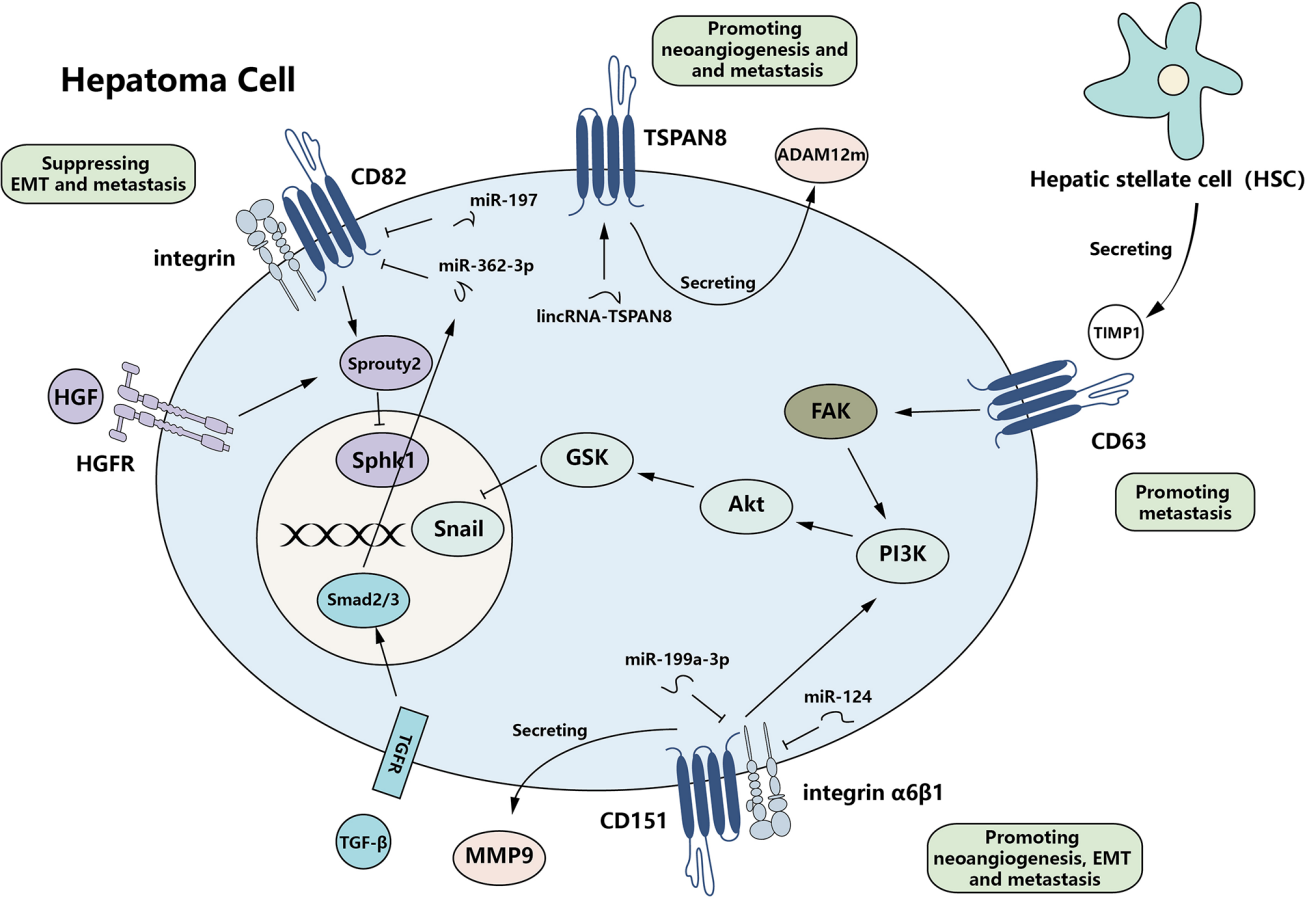
Complexity of the function of tetraspanins in HCC metastasis. Tetraspanins can bidirectionally regulate HCC metastasis by affecting EMT-related protein expression and invasiveness/migration. CD63, CD151, and TSPAN8 serve as promoters of metastasis, while CD82 functions as a repressor. Multiple signaling pathways are active and finally regulate the expression of downstream effectors. Meanwhile, various non-coding RNAs can regulate the expression of tetraspanins. HCC, hepatocellular carcinoma; EMT, epithelial–mesenchymal transition.

The members of tetraspanin family can bidirectionally regulate HCC progression. Some members, such as CD151 and TSPAN8 (50, 51), can enhance the invasiveness and mobility of HCC cells, while the metastasis-suppressive effect of some tetraspanins is also evident in various kinds of tumors, including HCC (52). Tetraspanins also function in epithelial–mesenchymal transition (EMT), the process of which contributes to both the initiation and metastasis of HCC. Generally speaking, tetraspanins are involved in multiple steps of hepatoma metastasis (**Figure 2**).

### 5.1 Role of Tetraspanin in HCC and Epithelial–Mesenchymal Transition

EMT plays an important role in the initiation and progression of the tumor. In HCC, CD82 and CD151 are often associated with EMT.

#### 5.1.1 CD82

It has been reported that CD82 can negatively regulate EMT in several other human cancers, such as non-small cell lung cancer and prostate cancer (53–57). In HCC cells, the levels of CD82 are also correlated with those of EMT markers. When CD82 is reduced through the TGF-β signaling pathway, the level of epithelial marker E-cadherin is also decreased while the level of mesenchymal marker N-cadherin is elevated, which means EMT progression is positively regulated (58).

#### 5.1.2 CD151

CD151 is upregulated in human HCC and various other cancers (59, 60) and exerts a positive effect on EMT (61, 62). CD151 complexes with integrin α6β1 and amplifies α6β1 signaling, which can activate the PI3K signaling pathway and then induce EMT (62). Sterk et al. found in the human erythroleukemic cell line, CD151, regulates α6β1 signals by directly interacting with the α subunits of integrins (63), but whether it employs the same mechanism in HCC cells still needs further research. The association between CD151 and α6 integrin can affect the mode of diffusion of α6 integrins and eventually supports their function, such as adhesion (64).

Since CD151 can favor EMT in HCC cells *via* the PI3K/Akt signaling pathway, Zhang et al. also considered the activation of this pathway relating to sorafenib resistance of HCC cells (61). They found that in high-CD151 expression HCC cells, the sensitivity to sorafenib is inversely correlated to CD151 levels. These findings provide a factual theoretical basis for clinical medication. Clinicians may need to consider whether the expression level of CD151 would influence the chemotherapy plans of HCC patients.

### 5.2 Role of Tetraspanin in HCC Invasiveness and Migration

The suppressive function of CD82 in metastasis has been validated in various kinds of tumors (65, 66), which means that the downregulation of CD82 can be a characteristic of metastatic tumors (5, 13). Approximately 20 years ago, the role of CD82 in hepatoma metastasis and prognosis has been reported (67, 68). The levels of CD82 are significantly reduced in cancerous tissues of HCC patients, especially in metastatic tumors and satellite metastases (68). This negative relationship between CD82 levels in HCC cells and their invasiveness ability has also proven both *in vitro* and *in vivo* (52, 69).

#### 5.2.1 CD82

Co-culture of HCC cells and vein endothelial cells suggests that CD82 may mediate the communication between cancer cells and endothelial cells (70). However, this study was conducted *in vitro* and was based on cells from two different species and thus may not elucidate the real mechanism in the human body. Through cell biomechanics analysis, measuring the visco-elastic properties of HCC tissues using the micropipette aspiration technique, Yang et al. showed that the adhesion force was significantly higher in CD82 upregulated cell lines compared with control. Meanwhile, the interaction between HCC cells and the extracellular matrix also proved to be enhanced in the xenografted and orthotopic tumors (69). All these results show that CD82 can strengthen the cell–cell and cell–matrix adhesion of HCC cells.

CD82 can suppress metastasis *via* regulating cell signaling pathways such as EGFR signaling pathway, HGFR signaling pathway, and the Wnt-β catenin signaling pathway (5, 13). In HCC, it is clear that CD82 can influence the HGFR pathway (71). CD82 can induce Sprouty2 expression, which acts as a negative regulator of the HGFR pathway (72). The upregulation of Sprouty2 ultimately decreases the activity of SphK1, a vital intracellular second messenger transducing growth factor bioactivity (73). Another suggested mechanism involving CD82 suppression of tumor metastasis is the interaction of CD82 and integrin. It is concomitant with decreased adhesion ability (5). However, since it is found in the other tumor, their relationship in HCC still needs further research.

The levels of CD82 expression are influenced by multiple factors. MiR-197 can bind to the 3′-UTR of the CD82 gene and subsequently inhibits the transcription of CD82 mRNA. qRT-PCR analysis shows that miR-197 is overexpressed in the cancerous tissue of HCC patients (74). Besides, CD82 is the target gene of p53. Acetylation and overexpression of p53 favor the transcription of CD82 (75). All these molecules influencing the expression of CD82 might be involved in the progression of HCC.

#### 5.2.2 CD151

CD151 is reported to be the first member of the tetraspanin family associated with the promotion of metastasis (76). It plays a pro-metastatic role in multiple cancers (49, 77). The role played by CD151 in HCC has been elucidated in detail from laboratory studies to the effects on clinical prognosis. All these studies suggest that the upregulation of CD151 might be a sensitive predictor of HCC metastasis (50, 78).

Devbhandari et al. showed that CD151 might induce metastasis through an integrin β1-dependent manner (78). Meanwhile, CD151 can induce MMP9 expression and facilitate extracellular matrix degradation and migration of cancer cells, which all contribute to the metastasis of HCC (50).

The crosstalk of CD151 with its upstream effectors is relatively complicated. It is reported that several non-coding RNAs can influence the level of CD151 and, ulteriorly, the metastasis of HCC (**Table 1**). Kim et al. showed that miR-199a-3p could impair the expression of CD151 in HCC cell lines and finally reduce cell migration and invasion *in vitro* (79). MiR-124 also targets CD151 mRNA, serving as the suppressor of HCC cell migration and invasion, sharing a similar effect with miR-199a-3p (80). Further studies suggest that some competing endogenous RNAs (ceRNAs), such as the mRNAs of oncogene PIK3C2A and laminin γ1 LAMC1, are able to bind miR-124 competitively, which reduces the level of miR-124 and then increases the level of CD151 (79, 80).

**Table 1:** The non-coding RNAs that regulate the expression of tetraspanin.

	Non-coding RNAs regulating tetraspanin expression	Reference
CD82	MiR-197 (74)	(74)
CD151	MiR-199a-3p (79), miR-124 (80), miR-128 (61)	(61, 79, 80)
TSPAN8	LincRNA-TSPAN8 (81)	(81)

Based on a previous study indicating that CD151 interacts with integrin β1, Ke et al. generated monoclonal antibody recognizing the integrin β1 binding site of CD151 (82). Consistent with its function in angiogenesis, invasiveness, and migration, the blockade of CD151 suppresses both vascular remodeling and metastasis both *in vitro* and *in vivo*. While the HCC cell proliferation was not slowed down *in vitro*, tumor growth inhibition may have been due to inadequate nutrition resulting from its anti-angiogenic effect. No further details about the antibody are currently available. Further studies are needed to assess the efficacy and safety of this antibody for metastatic HCC before it can be put into clinical use.

#### 5.2.3 TSPAN8

As in other tumors such as pancreatic cancer and breast cancer (83–85), TSPAN8 is upregulated at both the mRNA and protein levels, and its level is associated with the potential of the tumor to metastasize (85, 86). Overexpression of TSPAN8 *in vitro* demonstrates that the level of TSPAN8 is not correlated with the potential of proliferation and adhesion, but it is confirmed that its level is positively correlated with intrahepatic metastasis (46, 51, 87). However, Akiel et al. and Fang et al. suggested that TSPAN8 may increase the tumor size by facilitating neoangiogenesis (46), which is contrary to results by Kanetaka et al., indicating that TSPAN8 would not influence the size of the primary tumor (51). This contradiction may be owing to the weak and indirect growth-promoting effects of TSPAN8. Larger tumor size might be a side effect of neoangiogenesis. Thus, the correlation between TSPAN8 level and tumor size is not obvious.

An *in vitro* study showed that TSPAN8 may serve as a promoter of metastasis in HCC cells by inducing ADAM12m, a type of matrix metalloproteinase; and its expression may enhance the degradation of the extracellular matrix (87). It is found that in the *Drosophila*, tetraspanins can directly interact with ADAM10, control its trafficking by regulating its exit from endoplasmic reticulum, and finally decide ADAM10 expression on the surface (88). Maybe in human HCC, TSPAN8 would regulate ADAM12m expression in a similar way. TSPAN8 and α6β4 integrin can form a complex, which is internalized and then re-expressed, acting to ultimately decrease the adhesion to laminin 5 and shifting from an adhesion-supporting stage into a migration-supporting stage in pancreatic adenocarcinoma (84, 89). In resting stage, the complex of TSPAN8 and integrin localize at the trail of cancer cell; but under PKC signaling simulation, the complex will distribute in a different way. This mechanism may also contribute to the metastasis of HCC cells, although no evidence has been found.

It is reported that in HCC the expression of TSPAN8 is regulated by astrocyte elevated gene-1 (AEG-1), an oncogene upregulated in various cancers; and TSPAN8 acts as one essential effector on its pro-metastasis effect (46). Besides, lincRNA-TSPAN8 also leads to a high expression of TSPAN8 (81) (**Table 1**).

Despite the fact that TSPAN8 has not been an available therapeutic target in HCC, there are several antibodies of TSPAN8 that may act as candidates for potential agents for colorectal cancer (CRC) and ovarian cancer. In recent years, Kim et al. found that the TSPAN8 large extracellular loop (TSPAN8-LEL) is a critical domain of its function, and the authors developed a new type of antibody, using phage display technology, targeting TSPAN8-LEL (90). This anti-TSPAN8 antibody has been proven to suppress the invasiveness of HCT116 and LoVo CRC cell lines. Meanwhile, according to the relatively specific distribution of TSPAN8, a radiolabeled anti-TSPAN8 antibody was developed, which could inhibit tumor growth significantly in nude mice carrying HT29 tumors (91). In ovarian cancer, TSPAN8-LEL also performs similar functions in tumor metastasis. TSPAN8-LEL recognized antibody shows inhibition of ovarian cancer cell metastasis both *ex vivo* and *in vivo* without severe *in vitro* cytotoxicity or *in vivo* nephrotoxicity and hepatotoxicity (92). Although in different types of cancers tetraspanins may interact with different partners and function discordantly, with the consistent role of TSPAN8 and its upregulation in HCC, it may also be a promising therapeutic target *via* a highly specific antibody.

#### 5.2.4 Other Tetraspanins

CD9 and CD63 also act as pro-metastatic effectors of HCC. In highly metastatic HCC cell lines, CD9 is upregulated, and inhibition of CD9 expression can reduce its migration and invasion (93). It is believed that CD63 can mediate the TGF-β signaling pathway through binding of inhibitors of metalloproteinase-1 (TIMP-1), a key mediator of TGF-β-mediated crosstalk between hepatic stellate cells (HSCs) and HCC cells, activate the FAK-Akt signaling pathway in HCC cells, and further favor migration and survival of HCC cells (31). TSPAN7, besides its growth-inhibitory effect, Qi et al. also found it acted as a suppressor of metastasis (44). CD81 may be associated with organ-specific metastasis. It has been reported that the level of CD81 mRNA is downregulated in high lymphatic metastatic potential HCC cell lines compared with high lung metastatic potential HCC cell lines, which suggest that some certain tetraspanins might impose a potential impact on the target of HCC metastasis (81).

## 6 Role of Tetraspanin in Exosomes in HCC

Exosome, a nanosized vesicle secreted by both tumor cells and normal cells, often contains proteins, RNA, and even DNA from its host cell. Since it can carry cargos and information between different kinds of cells, it is regarded as a novel intercellular communication mechanism. Exosomes take part in nearly all the processes of the development of HCC such as hepatocarcinogenesis, HCC growth, angiogenesis, and metastasis (94). Tetraspanins are also proven to play a key role in exosome formation, target selection, and uptake in various types of tissues and cancers (95). Current studies have already shown that exosomal tetraspanins are an important marker for HCC; and tetraspanins such as CD9, CD31, and CD63 were used as collection markers of exosomes derived from HCC cells (96–101). Thus, it is reasonable to believe that in HCC, tetraspanins are also involved in the process of exosome formation and function, although there are still few direct evidences.

Since exosomes can be secreted by HCC cells into peripheral blood, exosomal tetraspanins have the potential to be used as a novel diagnostic target for early detection or treatment monitor of HCC. In other cancers, such as oral cancer, exosomal tetraspanins CD9, CD63, and CD81 can be potential biomarkers for early diagnosis in high-risk patients before any clinical symptoms (102). It is worthwhile to investigate the sensitivity and specificity of these tetraspanin serum levels in HCC early diagnosis or treatment monitoring in the future.

## 7 Correlations of Tetraspanin With HCC Clinicopathological Parameter and Patient Prognosis

As many tetraspanins are associated with HCC initiation and progression, the expression of some specific tetraspanins can also help to diagnose HCC and predict HCC patients’ prognosis. Based on the available evidence, CD81 and CD82, and TSPAN6 can serve as cancer suppressors, while CD151, TSPAN1, and TSPAN8 act as cancer promoters in the diagnosis and prognosis of HCC patients (50, 68, 78, 86, 87, 103–105) (**Tables 2** and **3**).

**Table 2:** The expression level of tetraspanins and their relationship with tumor characteristics.

	Expression		Tumor characteristics				Reference
	Expression level in HCC tissue	Expression level in serum	Tumor size	Tumor number	Tumor encapsulation	Differential grading	
CD81			NS (104)			Negative (*) (103)	(103, 104)
CD82 (KAI1)	Downregulated (**, compared with adjacent cancerous tissues and distant cancerous tissues) (105); downregulated (*, compared with adjacent non-tumorous tissues) (86)	Downregulated (**, compared with cirrhosis, hepatitis, and normal control samples) (105)	NS (68, 105)			NS (68, 105)	(68, 86, 105)
CD151	Upregulated (*, compared with adjacent non-tumorous tissues) (50)		Positive (*) (50, 78)	Positive (**) (50) (*) (78)	NS (50, 78)	Positive (*) (50); NS (78)	(50, 78)
TSPAN1	Upregulated (**, compared with both adjacent non-tumorous tissues and normal tissues from control group) (106); upregulated (*, compared with adjacent non-tumorous tissues) (37)					Positive (*) (106)	(37, 106)
TSPAN8 (CO-029)	Upregulated (*, compared with adjacent non-tumorous tissues) (85, 86)		NS (86, 87)	NS (87)	NS (87)	Positive (**) (86) (*) (87)	(85–87)

For tumor characteristics, positive means HCC malignancy is positively correlated with the level of tetraspanins.

HCC, hepatocellular carcinoma; NS, not significant.

*p < 0.05.

**p < 0.01.

**Table 3:** The relationship between the level of tetraspanins and tumor metastasis, patient prognosis.

	Metastasis				Prognosis		Reference
	TMN stage	Intrahepatic metastasis	Vascular metastasis	Lymph node or peritoneal metastasis	Overall survival rate	Cumulative recurrence	
CD81			NS (104)	Negative (*) (104)	NS (multivariate analysis) (104)		(104)
CD82 (KAI1)	Negative (*) (68, 105)	Negative (*) (68) (**) (105)	Negative (**) (105)	Negative (*) (68) (**) (105)			(68, 105)
CD151	Positive (**) (50) (*) (78)		Positive (**) (50) (*) (78)		Negative (**, univariate analysis; *, multivariate analysis) (50) (*, univariate analysis; NS, multivariate analysis) (78)	Positive (**, univariate analysis; *, multivariate analysis) (50) (**, univariate analysis; *, multivariate analysis) (78)	(50, 78)
TSPAN1	Positive (**) (38)			Positive (*) (106)	Negative (*, univariate analysis; *, multivariate analysis) (106)		(38, 106)
TSPAN8 (CO-029)	Positive (*) (87)	Positive (*) (86)	Positive (*) (87)		Negative (*, univariate analysis; *, multivariate analysis) (87)		(86, 87)

For metastasis, positive means HCC malignancy is positively correlated with the level of tetraspanins. For prognosis, positive means the overall survival rate and cumulative recurrence rate are positively correlated with the level of tetraspanins.

NS, not significant.

*p < 0.05.

**p < 0.01.

Few studies have investigated the function of TSPAN6 in HCC; until recently, a study suggests TSPAN6 may associate with the aggregates of CD20+ B cells in tumor. It is also shown that the expression of TSPAN8 positively correlates with a better prognosis (107). However, we still know little about the function of TSPAN6.

## 8 Conclusions and Prospects

Tetraspanins interact with other multiple molecules and form a complicated and vast network participating in numerous essential cell activities. As discussed above, tetraspanins have been shown to be involved in the entire life cycle of HCC. Since the tetraspanin family plays a bidirectional function at each stage of carcinogenesis, the blockade of cancer-promoting tetraspanins or their downstream factors may be successful therapeutic targets in the future. To date, specific antibodies or RNA interference has been used to block tetraspanin-related signals. Some reagents have achieved significant results in animal studies (82). However, as for clinical use, there is still a long way to go. For the tetraspanins that serve as suppressors, some agonists may be used to amplify the protective signals mediated by, for instance, CD82.

Sorafenib, a vascular endothelial growth factor (VEGF) inhibitor, is the first-line medication of HCC. Sorafenib monotherapy has become the gold standard for systemic treatment of advanced HCC. Since it has been proven that sorafenib resistance is strongly correlated with the levels of CD151 in HCC cells, CD151 and its downstream pathway might be the key to combat antineoplastic drug resistance. However, how CD151 attenuates sorafenib resistance still needs further research. As high CD151 levels predict high malignancy and low sorafenib sensitivity simultaneously, this underlines the substantial heterogeneity between high and low CD151 levels in HCC and the importance of assessing the molecular type of tumor when choosing a treatment regimen.

Nevertheless, there is no perfect treatment regimen for HCC. As more detailed information about the relationship between tetraspanins and hepatoma becomes available, we hope that the tetraspanin family can be a promising anticancer target that provides an approach for curing HCC.

## Author Contributions

SC and YD designed and wrote all the parts of the manuscript. HP and JS supervised and revised the manuscript. All authors contributed to the article and approved the submitted version.

## Funding

This study was conducted with the support by the National Natural Science Foundation of China (Grant No. 82073400).

## Conflict of Interest

The authors declare that the research was conducted in the absence of any commercial or financial relationships that could be construed as a potential conflict of interest.

## Publisher’s Note

All claims expressed in this article are solely those of the authors and do not necessarily represent those of their affiliated organizations, or those of the publisher, the editors and the reviewers. Any product that may be evaluated in this article, or claim that may be made by its manufacturer, is not guaranteed or endorsed by the publisher.

## Abbreviations

HCC, hepatocellular carcinoma; TM, transmembrane; ECL, extracellular loop; ADAM10, A Disintegrin And Metalloprotease Domain 10; HSECs, hepatic sinusoidal endothelial cells; VCAM-1, Vascular Cell Adhesion Molecule-1; CTGF, connective tissue growth factor; EpCAM, epithelial cell adhesion molecule; CK19, cytokeratin-19; YAP, Yes-associated Protein; CREB, cAMP-response element-binding protein; KLF4, Krüppel-like factor 4; NET-1, neuroepithelial transforming gene 1; GEF, guanine nucleotide exchange factor; CDK4, cyclin-dependent kinase 4; Rb, retinoblastoma; MMP9, metalloproteinase 9; GPCRs, G protein-coupled receptors; TEMs, membrane microdomains; HGFR, hepatocyte growth factor receptor; EGFR, epidermal growth factor receptor; TGF-β, transforming growth factor-β; HUVECs, human umbilical vein endothelial cells; EMT, epithelial–mesenchymal transition; ceRNAs, competing endogenous RNAs; ADAM12m, A Disintegrin And Metalloproteinase Domain 12; AEG-1, astrocyte elevated gene-1; CRC, colorectal cancer; TSPAN-LEL, TSPAN8 large extracellular loop; TIMP-1, metalloproteinase-1; HSCs, hepatic stellate cells; VEGF, vascular endothelial growth factor; NS, not significant.

## References

[1] Bray F Ferlay J Soerjomataram I Siegel RL Torre LA Jemal A. Global Cancer Statistics 2018: GLOBOCAN Estimates of Incidence and Mortality Worldwide for 36 Cancers in 185 Countries. CA: Cancer J Clin (2018) 68(6):394–424. 10.3322/caac.21492 30207593

[2] Ferlay J Soerjomataram I Dikshit R Eser S Mathers C Rebelo M. Cancer Incidence and Mortality Worldwide: Sources, Methods and Major Patterns in GLOBOCAN 2012. Int J Cancer (2015) 136(5):E359–86. 10.1002/ijc.29210 25220842

[3] Davila JA Morgan RO Shaib Y McGlynn KA El-Serag HB. Hepatitis C Infection and the Increasing Incidence of Hepatocellular Carcinoma: A Population-Based Study. Gastroenterology (2004) 127(5):1372–80. 10.1053/j.gastro.2004.07.020 15521006

[4] Maucort-Boulch D de Martel C Franceschi S Plummer M. Fraction and Incidence of Liver Cancer Attributable to Hepatitis B and C Viruses Worldwide. Int J Cancer (2018) 142(12):2471–7. 10.1002/ijc.31280 29388206

[5] Zoller M. Tetraspanins: Push and Pull in Suppressing and Promoting Metastasis. Nat Rev Cancer (2009) 9(1):40–55. 10.1038/nrc2543 19078974

[6] Hemler ME. Tetraspanin Functions and Associated Microdomains. Nat Rev Mol Cell Biol (2005) 6(10):801–11. 10.1038/nrm1736 16314869

[7] Huang S Yuan S Dong M Su J Yu C Shen Y. The Phylogenetic Analysis of Tetraspanins Projects the Evolution of Cell-Cell Interactions From Unicellular to Multicellular Organisms. Genomics (2005) 86(6):674–84. 10.1016/j.ygeno.2005.08.004 16242907

[8] Charrin S Jouannet S Boucheix C Rubinstein E. Tetraspanins at a Glance. J Cell Sci (2014) 127(Pt 17):3641–8. 10.1242/jcs.154906 25128561

[9] Hemler ME. Specific Tetraspanin Functions. J Cell Biol (2001) 155(7):1103–7. 10.1083/jcb.200108061 PMC2199333 11756464

[10] Boucheix C Rubinstein E. Tetraspanins. Cell Mol Life sciences: CMLS (2001) 58(9):1189–205. 10.1007/PL00000933 PMC11337403 11577978

[11] Jiang X Zhang J Huang Y. Tetraspanins in Cell Migration. Cell adhesion migration (2015) 9(5):406–15. 10.1080/19336918.2015.1005465 PMC4955372 26091149

[12] Yang YG Sari IN Zia MF Lee SR Song SJ Kwon HY. Tetraspanins: Spanning From Solid Tumors to Hematologic Malignancies. Exp Hematol (2016) 44(5):322–8. 10.1016/j.exphem.2016.02.006 26930362

[13] Hemler ME. Tetraspanin Proteins Promote Multiple Cancer Stages. Nat Rev Cancer (2014) 14(1):49–60. 10.1038/nrc3640 24505619

[14] Sala-Valdes M Ailane N Greco C Rubinstein E Boucheix C. Targeting Tetraspanins in Cancer. Expert Opin Ther Targets (2012) 16(10):985–97. 10.1517/14728222.2012.712688 22880813

[15] Pileri P Uematsu Y Campagnoli S Galli G Falugi F Petracca R. Binding of Hepatitis C Virus to CD81. Sci (New York NY) (1998) 282(5390):938–41. 10.1126/science.282.5390.938 9794763

[16] Lammerding J Kazarov AR Huang H Lee RT Hemler ME. Tetraspanin CD151 Regulates Alpha6beta1 Integrin Adhesion Strengthening. Proc Natl Acad Sci USA (2003) 100(13):7616–21. 10.1073/pnas.1337546100 PMC164635 12805567

[17] Hong IK Jeoung DI Ha KS Kim YM Lee H. Tetraspanin CD151 Stimulates Adhesion-Dependent Activation of Ras, Rac, and Cdc42 by Facilitating Molecular Association Between β1 Integrins and Small GTPases. J Biol Chem (2012) 287(38):32027–39. 10.1074/jbc.M111.314443 PMC3442534 22843693

[18] Sadej R Grudowska A Turczyk L Kordek R Romanska HM. CD151 in Cancer Progression and Metastasis: A Complex Scenario. Lab Invest; J Tech Methods Pathol (2014) 94(1):41–51. 10.1038/labinvest.2013.136 24247563

[19] Chen CJ Yang HI Su J Jen CL You SL Lu SN. Risk of Hepatocellular Carcinoma Across a Biological Gradient of Serum Hepatitis B Virus DNA Level. JAMA (2006) 295(1):65–73. 10.1001/jama.295.1.65 16391218

[20] Yu G Bing Y Li W Xia L Liu Z. Hepatitis B Virus Inhibits the Expression of CD82 Through Hypermethylation of Its Promoter in Hepatoma Cells. Mol Med Rep (2014) 10(5):2580–6. 10.3892/mmr.2014.2495 25119390

[21] Huang YT Jen CL Yang HI Lee MH Su J Lu SN. Lifetime Risk and Sex Difference of Hepatocellular Carcinoma Among Patients With Chronic Hepatitis B and C. J Clin Oncol: Off J Am Soc Clin Oncol (2011) 29(27):3643–50. 10.1200/jco.2011.36.2335 PMC4874144 21859997

[22] Zhang S Kodys K Babcock GJ Szabo G. CD81/CD9 Tetraspanins Aid Plasmacytoid Dendritic Cells in Recognition of Hepatitis C Virus-Infected Cells and Induction of Interferon-Alpha. Hepatol (Baltimore Md) (2013) 58(3):940–9. 10.1002/hep.25827 PMC4511847 22577054

[23] Poynard T Mathurin P Lai CL Guyader D Poupon R Tainturier MH. A Comparison of Fibrosis Progression in Chronic Liver Diseases. J Hepatol (2003) 38(3):257–65. 10.1016/s0168-8278(02)00413-0 12586290

[24] Friedman SL. Evolving Challenges in Hepatic Fibrosis. Nat Rev Gastroenterol Hepatol (2010) 7(8):425–36. 10.1038/nrgastro.2010.97 20585339

[25] Jouannet S Saint-Pol J Fernandez L Nguyen V Charrin S Boucheix C. TspanC8 Tetraspanins Differentially Regulate the Cleavage of ADAM10 Substrates, Notch Activation and ADAM10 Membrane Compartmentalization. Cell Mol Life Sciences: CMLS (2016) 73(9):1895–915. 10.1007/s00018-015-2111-z PMC4819958 26686862

[26] Lubecka K Flower K Beetch M Qiu J Kurzava L Buvala H. Loci-Specific Differences in Blood DNA Methylation in HBV-Negative Populations at Risk for Hepatocellular Carcinoma Development. Epigenetics (2018) 13(6):605–26. 10.1080/15592294.2018.1481706 PMC6140905 29927686

[27] Wadkin JCR Patten DA Kamarajah SK Shepherd EL Novitskaya V Berditchevski F. CD151 Supports VCAM-1-Mediated Lymphocyte Adhesion to Liver Endothelium and Is Upregulated in Chronic Liver Disease and Hepatocellular Carcinoma. Am J Physiol Gastrointest Liver Physiol (2017) 313(2):G138–49. 10.1152/ajpgi.00411.2016 PMC5582880 28473332

[28] Sidahmed-Adrar N Ottavi JF Benzoubir N Ait Saadi T Bou Saleh M Mauduit P. Tspan15 Is a New Stemness-Related Marker in Hepatocellular Carcinoma. Proteomics (2019) 19(21-22):e1900025. 10.1002/pmic.201900025 31390680

[29] Ma L Wang J Lin J Pan Q Yu Y Sun F. Cluster of Differentiation 166 (CD166) Regulated by Phosphatidylinositide 3-Kinase (PI3K)/AKT Signaling to Exert Its Anti-Apoptotic Role *via* Yes-Associated Protein (YAP) in Liver Cancer. J Biol Chem (2014) 289(10):6921–33. 10.1074/jbc.M113.524819 PMC3945353 24482231

[30] Wang J Zhou Y Li D Sun X Deng Y Zhao Q. TSPAN31 Is a Critical Regulator on Transduction of Survival and Apoptotic Signals in Hepatocellular Carcinoma Cells. FEBS Lett (2017) 591(18):2905–18. 10.1002/1873-3468.12737 28670683

[31] Park SA Kim MJ Park SY Kim JS Lim W Nam JS. TIMP-1 Mediates TGF-Beta-Dependent Crosstalk Between Hepatic Stellate and Cancer Cells *via* FAK Signaling. Sci Rep (2015) 5:16492. 10.1038/srep16492 26549110 PMC4637930

[32] Ramalho-Santos M Yoon S Matsuzaki Y Mulligan RC Melton DA. “Stemness”: Transcriptional Profiling of Embryonic and Adult Stem Cells. Sci (New York NY) (2002) 298(5593):597–600. 10.1126/science.1072530 12228720

[33] Gilsanz A Sanchez-Martin L Gutierrez-Lopez MD Ovalle S Machado-Pineda Y Reyes R. ALCAM/CD166 Adhesive Function Is Regulated by the Tetraspanin CD9. Cell Mol Life Sciences: CMLS (2013) 70(3):475–93. 10.1007/s00018-012-1132-0 PMC11113661 23052204

[34] Wang J Ma L Weng W Qiao Y Zhang Y He J. Mutual Interaction Between YAP and CREB Promotes Tumorigenesis in Liver Cancer. Hepatol (Baltimore Md) (2013) 58(3):1011–20. 10.1002/hep.26420 23532963

[35] Li Y Yu S Li L Chen J Quan M Li Q. KLF4-Mediated Upregulation of CD9 and CD81 Suppresses Hepatocellular Carcinoma Development *via* JNK signaling. Cell Death Dis (2020) 11(4):299. 10.1038/s41419-020-2479-z 32350244 PMC7190708

[36] Murray D Horgan G Macmathuna P Doran P. NET1-Mediated RhoA Activation Facilitates Lysophosphatidic Acid-Induced Cell Migration and Invasion in Gastric Cancer. Br J Cancer (2008) 99(8):1322–9. 10.1038/sj.bjc.6604688 PMC2570507 18827818

[37] Chen L Wang Z Zhan X Li DC Zhu YY Zhu J. Association of NET-1 Gene Expression With Human Hepatocellular Carcinoma. Int J Surg Pathol (2007) 15(4):346–53. 10.1177/1066896907306083 17913940

[38] Shen SQ Li K Zhu N Nakao A. Expression and Clinical Significance of NET-1 and PCNA in Hepatocellular Carcinoma. Med Oncol (Northwood London England) (2008) 25(3):341–5. 10.1007/s12032-008-9042-6 18214716

[39] Wu B Qiao Q Han X Jing H Zhang H Liang H. Targeted Nanobubbles in Low-Frequency Ultrasound-Mediated Gene Transfection and Growth Inhibition of Hepatocellular Carcinoma Cells. Tumour Biol (2016) 37(9):12113–21. 10.1007/s13277-016-5082-2 27216880

[40] Shang H Wu B Liang X Sun Y Han X Zhang L. Evaluation of Therapeutic Effect of Targeting Nanobubbles Conjugated With NET-1 siRNA by Shear Wave Elastography: An *In Vivo* Study of Hepatocellular Carcinoma Bearing Mice Model. Drug Deliv (2019) 26(1):944–51. 10.1080/10717544.2019.1667450 PMC6764407 31544556

[41] Sun X Wang M Zhang F Kong X. Inhibition of NET-1 Suppresses Proliferation and Promotes Apoptosis of Hepatocellular Carcinoma Cells by Activating the PI3K/AKT Signaling Pathway. Exp Ther Med (2019) 17(3):2334–40. 10.3892/etm.2019.7211 PMC6395973 30867719

[42] Wu B Shang H Liang X Sun Y Jing H Han X. Preparation of Novel Targeting Nanobubbles Conjugated With Small Interfering RNA for Concurrent Molecular Imaging and Gene Therapy *In Vivo*. FASEB J Off Publ Fed Am Societies Exp Biol (2019) 33(12):14129–36. 10.1096/fj.201900716RR 31657628

[43] Wu B Shang H Liu J Liang X Yuan Y Chen Y. Quantitative Proteomics Analysis of FFPE Tumor Samples Reveals the Influences of NET-1 siRNA Nanoparticles and Sonodynamic Therapy on Tetraspanin Protein Involved in HCC. Front Mol Biosci (2021) 8:678444. 10.3389/fmolb.2021.678444 34041269 PMC8141748

[44] Qi Y Li H Lv J Qi W Shen L Liu S. Expression and Function of Transmembrane 4 Superfamily Proteins in Digestive System Cancers. Cancer Cell Int (2020) 20(1):314. 10.1186/s12935-020-01353-1 32694936 PMC7364658

[45] Takeda Y Kazarov AR Butterfield CE Hopkins BD Benjamin LE Kaipainen A. Deletion of Tetraspanin Cd151 Results in Decreased Pathologic Angiogenesis *In Vivo* and *In Vitro*. Blood (2007) 109(4):1524–32. 10.1182/blood-2006-08-041970 PMC1794066 17023588

[46] Akiel MA Santhekadur PK Mendoza RG Siddiq A Fisher PB Sarkar D. Tetraspanin 8 Mediates AEG-1-Induced Invasion and Metastasis in Hepatocellular Carcinoma Cells. FEBS Lett (2016) 590(16):2700–8. 10.1002/1873-3468.12268 PMC4992437 27339400

[47] Viallard C Larrivée B. Tumor Angiogenesis and Vascular Normalization: Alternative Therapeutic Targets. Angiogenesis (2017) 20(4):409–26. 10.1007/s10456-017-9562-9 28660302

[48] Shi GM Ke AW Zhou J Wang XY Xu Y Ding ZB. CD151 Modulates Expression of Matrix Metalloproteinase 9 and Promotes Neoangiogenesis and Progression of Hepatocellular Carcinoma. Hepatol (Baltimore Md) (2010) 52(1):183–96. 10.1002/hep.23661 20578262

[49] Zijlstra A Lewis J Degryse B Stuhlmann H Quigley JP. The Inhibition of Tumor Cell Intravasation and Subsequent Metastasis *via* Regulation of *In Vivo* Tumor Cell Motility by the Tetraspanin CD151. Cancer Cell (2008) 13(3):221–34. 10.1016/j.ccr.2008.01.031 PMC3068919 18328426

[50] Ke AW Shi GM Zhou J Wu FZ Ding ZB Hu MY. Role of Overexpression of CD151 and/or C-Met in Predicting Prognosis of Hepatocellular Carcinoma. Hepatol (Baltimore Md) (2009) 49(2):491–503. 10.1002/hep.22639 19065669

[51] Kanetaka K Sakamoto M Yamamoto Y Takamura M Kanematsu T Hirohashi S. Possible Involvement of Tetraspanin CO-029 in Hematogenous Intrahepatic Metastasis of Liver Cancer Cells. J Gastroenterol Hepatol (2003) 18(11):1309–14. 10.1046/j.1440-1746.2003.03182.x 14535989

[52] Si SH Yang JM Peng ZH Luo YH Zhou P. Effects of KAI1 Gene on Growth and Invasion of Human Hepatocellular Carcinoma MHCC97-H Cells. World J Gastroenterol (2004) 10(14):2019–23. 10.3748/wjg.v10.i14.2019 PMC4572325 15237426

[53] Tennis MA Van Scoyk MM Freeman SV Vandervest KM Nemenoff RA Winn RA. Sprouty-4 Inhibits Transformed Cell Growth, Migration and Invasion, and Epithelial-Mesenchymal Transition, and Is Regulated by Wnt7A Through PPARgamma in Non-Small Cell Lung Cancer. Mol Cancer Res: MCR (2010) 8(6):833–43. 10.1158/1541-7786.Mcr-09-0400 PMC2888899 20501643

[54] Zhang QH Yao YL Wu XY Wu JH Gu T Chen L. Anti-miR-362-3p Inhibits Migration and Invasion of Human Gastric Cancer Cells by Its Target Cd82. Dig Dis Sci (2015) 60(7):1967–76. 10.1007/s10620-015-3563-6 25652145

[55] Zhou L Yu L Wu S Feng Z Song W Gong X. Clinicopathological Significance of KAI1 Expression and Epithelial-Mesenchymal Transition in non-Small Cell Lung Cancer. World J Surg Oncol (2015) 13:234. 10.1186/s12957-015-0657-8 26231404 PMC4522085

[56] Lee J Byun HJ Lee MS Jin YJ Jeoung D Kim YM. The Metastasis Suppressor CD82/KAI1 Inhibits Fibronectin Adhesion-Induced Epithelial-to-Mesenchymal Transition in Prostate Cancer Cells by Repressing the Associated Integrin Signaling. Oncotarget (2017) 8(1):1641–54. 10.18632/oncotarget.13767 PMC5352085 27926483

[57] Lee MS Lee J Kim YM Lee H. The Metastasis Suppressor CD82/KAI1 Represses the TGF-Beta 1 and Wnt Signalings Inducing Epithelial-to-Mesenchymal Transition Linked to Invasiveness of Prostate Cancer Cells. Prostate (2019) 79(12):1400–11. 10.1002/pros.23837 31212375

[58] Zhang Q Huang F Yao Y Wang J Wei J Wu Q. Interaction of Transforming Growth Factor-Beta-Smads/microRNA-362-3p/CD82 Mediated by M2 Macrophages Promotes the Process of Epithelial-Mesenchymal Transition in Hepatocellular Carcinoma Cells. Cancer Sci (2019) 110(8):2507–19. 10.1111/cas.14101 PMC6676115 31215741

[59] Donnenberg VS Zhang JJ Moravcikova E Meyer EM Lu H Carson CT. Antibody-Based Cell-Surface Proteome Profiling of Metastatic Breast Cancer Primary Explants and Cell Lines. Cytometry Part A: J Int Soc Anal Cytol (2018) 93(4):448–57. 10.1002/cyto.a.23300 29498809

[60] Yu Y Liang C Wang S Zhu J Miao C Hua Y. CD151 Promotes Cell Metastasis *via* Activating TGF-Beta1/Smad Signaling in Renal Cell Carcinoma. Oncotarget (2018) 9(17):13313–23. 10.18632/oncotarget.24028 PMC5862580 29568359

[61] Zhang PF Wang F Wu J Wu Y Huang W Liu D. LncRNA SNHG3 Induces EMT and Sorafenib Resistance by Modulating the miR-128/CD151 Pathway in Hepatocellular Carcinoma. J Cell Physiol (2019) 234(3):2788–94. 10.1002/jcp.27095 30132868

[62] Ke AW Shi GM Zhou J Huang XY Shi YH Ding ZB. CD151 Amplifies Signaling by Integrin Alpha6beta1 to PI3K and Induces the Epithelial-Mesenchymal Transition in HCC Cells. Gastroenterology (2011) 140(5):1629–41.e15. 10.1053/j.gastro.2011.02.008 21320503

[63] Sterk LM Geuijen CA van den Berg JG Claessen N Weening JJ Sonnenberg A. Association of the Tetraspanin CD151 With the Laminin-Binding Integrins Alpha3beta1, Alpha6beta1, Alpha6beta4 and Alpha7beta1 in Cells in Culture and In Vivo. J Cell Sci (2002) 115(Pt 6):1161–73. 10.1242/jcs.115.6.1161 11884516

[64] Yang XH Mirchev R Deng X Yacono P Yang HL Golan DE. CD151 Restricts the Alpha6 Integrin Diffusion Mode. J Cell Sci (2012) 125(Pt 6):1478–87. 10.1242/jcs.093963 PMC3336378 22328509

[65] Briese J Schulte HM Sajin M Bamberger C Redlin K Milde-Langosch K. Correlations Between Reduced Expression of the Metastasis Suppressor Gene KAI-1 and Accumulation of P53 in Uterine Carcinomas and Sarcomas. Virchows Archiv: An Int J Pathol (2008) 453(1):89–96. 10.1007/s00428-008-0608-7 18415123

[66] Protzel C Kakies C Kleist B Poetsch M Giebel J. Down-Regulation of the Metastasis Suppressor Protein KAI1/CD82 Correlates With Occurrence of Metastasis, Prognosis and Presence of HPV DNA in Human Penile Squamous Cell Carcinoma. Virchows Arch: An Int J Pathol (2008) 452(4):369–75. 10.1007/s00428-008-0590-0 18305955

[67] Sun HC Tang ZY Zhou G Li XM. KAI1 Gene Expression in Hepatocellular Carcinoma and Its Relationship With Intrahepatic Metastases. J Exp Clin Cancer Res: CR (1998) 17(3):307–11. 9894767

[68] Guo XZ Friess H Di Mola FF Heinicke JM Abou-Shady M Graber HU. KAI1, a New Metastasis Suppressor Gene, Is Reduced in Metastatic Hepatocellular Carcinoma. Hepatol (Baltimore Md) (1998) 28(6):1481–8. 10.1002/hep.510280606 9828210

[69] Yang JM Peng ZH Si SH Liu WW Luo YH Ye ZY. KAI1 Gene Suppresses Invasion and Metastasis of Hepatocellular Carcinoma MHCC97-H Cells *In Vitro* and in Animal Models. Liver Int: Off J Int Assoc Study Liver (2008) 28(1):132–9. 10.1111/j.1478-3231.2007.01620.x 18028322

[70] Takayama G Taniguchi A Okano T. Identification of Differentially Expressed Genes in Hepatocyte/Endothelial Cell Co-Culture System. Tissue Eng (2007) 13(1):159–66. 10.1089/ten.2006.0143 17518589

[71] Mu Z Wang H Zhang J Li Q Wang L Guo X. KAI1/CD82 Suppresses Hepatocyte Growth Factor-Induced Migration of Hepatoma Cells *via* Upregulation of Sprouty2. Sci China C Life Sci (2008) 51(7):648–54. 10.1007/s11427-008-0086-1 18622748

[72] Lee CC Putnam AJ Miranti CK Gustafson M Wang LM Vande Woude GF. Overexpression of Sprouty 2 Inhibits HGF/SF-Mediated Cell Growth, Invasion, Migration, and Cytokinesis. Oncogene (2004) 23(30):5193–202. 10.1038/sj.onc.1207646 15122328

[73] Maceyka M Payne SG Milstien S Spiegel S. Sphingosine Kinase, Sphingosine-1-Phosphate, and Apoptosis. Biochim Biophys Acta (2002) 1585(2-3):193–201. 10.1016/s1388-1981(02)00341-4 12531554

[74] Dai W Wang C Wang F Wang Y Shen M Chen K. Anti-miR-197 Inhibits Migration in HCC Cells by Targeting KAI 1/Cd82. Biochem Biophys Res Commun (2014) 446(2):541–8. 10.1016/j.bbrc.2014.03.006 24613834

[75] Zhao B Zhao W Wang Y Xu Y Xu J Tang K. Connexin32 Regulates Hepatoma Cell Metastasis and Proliferation *via* the P53 and Akt Pathways. Oncotarget (2015) 6(12):10116–33. 10.18632/oncotarget.2687 PMC4496344 25426556

[76] Tokuhara T Hasegawa H Hattori N Ishida H Taki T Tachibana S. Clinical Significance of CD151 Gene Expression in Non-Small Cell Lung Cancer. Clin Cancer Res: An Off J Am Assoc Cancer Res (2001) 7(12):4109–14. 11751509

[77] Sanjmyatav J Steiner T Wunderlich H Diegmann J Gajda M Junker K. A Specific Gene Expression Signature Characterizes Metastatic Potential in Clear Cell Renal Cell Carcinoma. J Urol (2011) 186(1):289–94. 10.1016/j.juro.2011.03.033 21600596

[78] Devbhandari RP Shi GM Ke AW Wu FZ Huang XY Wang XY. Profiling of the Tetraspanin CD151 Web and Conspiracy of CD151/integrin Beta1 Complex in the Progression of Hepatocellular Carcinoma. PloS One (2011) 6(9):e24901. 10.1371/journal.pone.0024901 21961047 PMC3178554

[79] Kim JH Badawi M Park JK Jiang J Mo X Roberts LR. Anti-Invasion and Anti-Migration Effects of miR-199a-3p in Hepatocellular Carcinoma Are Due in Part to Targeting CD151. Int J Oncol (2016) 49(5):2037–45. 10.3892/ijo.2016.3677 27599545

[80] Liu T Zu CH Wang SS Song HL Wang ZL Xu XN. PIK3C2A mRNA Functions as a miR-124 Sponge to Facilitate CD151 Expression and Enhance Malignancy of Hepatocellular Carcinoma Cells. Oncotarget (2016) 7(28):43376–89. 10.18632/oncotarget.9716 PMC5190030 27270320

[81] Fang TT Sun XJ Chen J Zhao Y Sun RX Ren N. Long non-Coding RNAs Are Differentially Expressed in Hepatocellular Carcinoma Cell Lines With Differing Metastatic Potential. Asian Pac J Cancer Prev: APJCP (2014) 15(23):10513–24. 10.7314/apjcp.2014.15.23.10513 25556502

[82] Ke AW Zhang PF Shen YH Gao PT Dong ZR Zhang C. Generation and Characterization of a Tetraspanin CD151/integrin Alpha6beta1-Binding Domain Competitively Binding Monoclonal Antibody for Inhibition of Tumor Progression in HCC. Oncotarget (2016) 7(5):6314–22. 10.18632/oncotarget.6833 PMC4868758 26756217

[83] Berthier-Vergnes O El Kharbili M de la Fouchardiere A Pointecouteau T Verrando P Wierinckx A. Gene Expression Profiles of Human Melanoma Cells With Different Invasive Potential Reveal TSPAN8 as a Novel Mediator of Invasion. Br J Cancer (2011) 104(1):155–65. 10.1038/sj.bjc.6605994 PMC3039798 21081927

[84] Gesierich S Paret C Hildebrand D Weitz J Zgraggen K Schmitz-Winnenthal FH. Colocalization of the Tetraspanins, CO-029 and CD151, With Integrins in Human Pancreatic Adenocarcinoma: Impact on Cell Motility. Clin Cancer Res: An Off J Am Assoc Cancer Res (2005) 11(8):2840–52. 10.1158/1078-0432.Ccr-04-1935 15837731

[85] Li J Chen X Zhu L Lao Z Zhou T Zang L. SOX9 Is a Critical Regulator of TSPAN8-Mediated Metastasis in Pancreatic Cancer. Oncogene (2021) 40(30):4884–93. 10.1038/s41388-021-01864-9 PMC8321899 34163029

[86] Kanetaka K Sakamoto M Yamamoto Y Yamasaki S Lanza F Kanematsu T. Overexpression of Tetraspanin CO-029 in Hepatocellular Carcinoma. J hepatology (2001) 35(5):637–42. 10.1016/s0168-8278(01)00183-0 11690710

[87] Fang T Lin J Wang Y Chen G Huang J Chen J. Tetraspanin-8 Promotes Hepatocellular Carcinoma Metastasis by Increasing ADAM12m Expression. Oncotarget (2016) 7(26):40630–43. 10.18632/oncotarget.9769 PMC5130032 27270327

[88] Dornier E Coumailleau F Ottavi JF Moretti J Boucheix C Mauduit P. TspanC8 Tetraspanins Regulate ADAM10/Kuzbanian Trafficking and Promote Notch Activation in Flies and Mammals. J Cell Biol (2012) 199(3):481–96. 10.1083/jcb.201201133 PMC3483123 23091066

[89] Herlevsen M Schmidt DS Miyazaki K Zoller M. The Association of the Tetraspanin D6.1A With the Alpha6beta4 Integrin Supports Cell Motility and Liver Metastasis Formation. J Cell Sci (2003) 116(Pt 21):4373–90. 10.1242/jcs.00760 13130099

[90] Kim TK Park CS Jeoung MH Lee WR Go NK Choi JR. Generation of a Human Antibody That Inhibits TSPAN8-Mediated Invasion of Metastatic Colorectal Cancer Cells. Biochem Biophys Res Commun (2015) 468(4):774–80. 10.1016/j.bbrc.2015.11.031 26562525

[91] Maisonial-Besset A Witkowski T Navarro-Teulon I Berthier-Vergnes O Fois G Zhu Y. Tetraspanin 8 (TSPAN 8) as a Potential Target for Radio-Immunotherapy of Colorectal Cancer. Oncotarget (2017) 8(13):22034–47. 10.18632/oncotarget.15787 PMC5400644 28423546

[92] Park CS Kim TK Kim HG Kim YJ Jeoung MH Lee WR. Therapeutic Targeting of Tetraspanin8 in Epithelial Ovarian Cancer Invasion and Metastasis. Oncogene (2016) 35(34):4540–8. 10.1038/onc.2015.520 26804173

[93] Lin Q Peng S Yang Y. Inhibition of CD9 Expression Reduces the Metastatic Capacity of Human Hepatocellular Carcinoma Cell Line MHCC97-H. Int J Oncol (2018) 53(1):266–74. 10.3892/ijo.2018.4381 29749468

[94] Chen R Xu X Tao Y Qian Z Yu Y. Exosomes in Hepatocellular Carcinoma: A New Horizon. Cell Commun Signal: CCS (2019) 17(1):1. 10.1186/s12964-018-0315-1 30616541 PMC6323788

[95] Malla RR Pandrangi S Kumari S Gavara MM Badana AK. Exosomal Tetraspanins as Regulators of Cancer Progression and Metastasis and Novel Diagnostic Markers. Asia Pac J Clin Oncol (2018) 14(6):383–91. 10.1111/ajco.12869 29575602

[96] Zou M You Y He S Wu XL. Effects of Hypoxic Exosomes on the Proliferation, Migration and Invasion of Hepatocellular Carcinoma Huh7 Cells. Zhonghua gan zang bing za zhi = Zhonghua ganzangbing zazhi = Chin J Hepatol (2019) 27(5):363–8. 10.3760/cma.j.issn.1007-3418.2019.05.008 PMC12769755 31177661

[97] Yang L Peng X Li Y Zhang X Ma Y Wu C. Long non-Coding RNA HOTAIR Promotes Exosome Secretion by Regulating RAB35 and SNAP23 in Hepatocellular Carcinoma. Mol Cancer (2019) 18(1):78. 10.1186/s12943-019-0990-6 30943982 PMC6446409

[98] Luo F Sun Z Han Q Xue C Bai C. Effect of Human Hepatocellular Carcinoma HepG2 Cell-Derived Exosome on the Differentiation of Mesenchymal Stem Cells and Their Interaction. Zhongguo yi xue ke xue yuan xue bao Acta Academiae Med Sinicae (2017) 39(3):312–7. 10.3881/j.issn.1000-503X.2017.03.003 28695799

[99] Qu Z Wu J Wu J Luo D Jiang C Ding Y. Exosomes Derived From HCC Cells Induce Sorafenib Resistance in Hepatocellular Carcinoma Both *In Vivo* and *In Vitro*. J Exp Clin Cancer Res: CR (2016) 35(1):159. 10.1186/s13046-016-0430-z 27716356 PMC5045585

[100] Ko SF Yip HK Zhen YY Lee CC Lee CC Huang CC. Adipose-Derived Mesenchymal Stem Cell Exosomes Suppress Hepatocellular Carcinoma Growth in a Rat Model: Apparent Diffusion Coefficient, Natural Killer T-Cell Responses, and Histopathological Features. Stem Cells Int (2015) 2015:853506. 10.1155/2015/853506 26345219 PMC4545422

[101] Tang Q Zhang X Zhang W Zhao S Chen Y. Identification and Characterization of Cell-Bound Membrane Vesicles. Biochim Biophys Acta Biomembranes (2017) 1859(5):756–66. 10.1016/j.bbamem.2017.01.013 28088446

[102] Zlotogorski-Hurvitz A Dayan D Chaushu G Salo T Vered M. Morphological and Molecular Features of Oral Fluid-Derived Exosomes: Oral Cancer Patients Versus Healthy Individuals. J Cancer Res Clin Oncol (2016) 142(1):101–10. 10.1007/s00432-015-2005-3 PMC11819233 26115960

[103] Inoue G Horiike N Onji M. The CD81 Expression in Liver in Hepatocellular Carcinoma. Int J Mol Med (2001) 7(1):67–71. 10.3892/ijmm.7.1.67 11115611

[104] Schoniger-Hekele M Hanel S Wrba F Muller C. Hepatocellular Carcinoma–Survival and Clinical Characteristics in Relation to Various Histologic Molecular Markers in Western Patients. Liver Int: Off J Int Assoc Study Liver (2005) 25(1):62–9. 10.1111/j.1478-3231.2004.0997.x 15698400

[105] Zhang W Zhao CG Sun HY Zheng WE Chen H. Expression Characteristics of KAI1 and Vascular Endothelial Growth Factor and Their Diagnostic Value for Hepatocellular Carcinoma. Gut Liver (2014) 8(5):536–42. 10.5009/gnl13331 PMC4164248 25071074

[106] Chen L Yuan D Wang GL Wang Y Wu YY Zhu J. Clinicopathological Significance of Expression of Tspan-1, Jab1 and P27 in Human Hepatocellular Carcinoma. J Korean Med Sci (2010) 25(10):1438–42. 10.3346/jkms.2010.25.10.1438 PMC2946652 20890423

[107] Bolimowska O Patten D Shetty S Berditchevski F O’Rourke J Cain O. TSPAN6: A Novel Player in the Microenvironment of Primary Liver Cancers. Gut (2021) 70(Suppl 1):A134–5. 10.1136/gutjnl-2020-bsgcampus.251

